# Characterization of 17α-hydroxysteroid dehydrogenase activity (17α-HSD) and its involvement in the biosynthesis of epitestosterone

**DOI:** 10.1186/1471-2091-6-12

**Published:** 2005-07-14

**Authors:** Véronique Bellemare, Frédérick Faucher, Rock Breton, Van Luu-The

**Affiliations:** 1 Oncology and Molecular Endocrinology Research Center Laval University Medical Center (CHUL) 2705 Laurier Boulevard Quebec, (Quebec) G1V 4G2, Canada

## Abstract

**Background:**

Epi-testosterone (epiT) is the 17α-epimer of testosterone. It has been found at similar level as testosterone in human biological fluids. This steroid has thus been used as a natural internal standard for assessing testosterone abuse in sports. EpiT has been also shown to accumulate in mammary cyst fluid and in human prostate. It was found to possess antiandrogenic activity as well as neuroprotective effects. So far, the exact pathway leading to the formation of epiT has not been elucidated.

**Results:**

In this report, we describe the isolation and characterization of the enzyme 17α-hydroxysteroid dehydrogenase. The name is given according to its most potent activity. Using cells stably expressing the enzyme, we show that 17α-HSD catalyzes efficienty the transformation of 4-androstenedione (4-dione), dehydroepiandrosterone (DHEA), 5α-androstane-3,17-dione (5α-dione) and androsterone (ADT) into their corresponding 17α-hydroxy-steroids : epiT, 5-androstene-3β,17α-diol (epi5diol), 5α-androstane-17α-ol-3-one (epiDHT) and 5α-androstane-3α,17α-diol (epi3α-diol), respectively. Similar to other members of the aldo-keto reductase family that possess the ability to reduce the keto-group into hydroxyl-group at different position on the steroid nucleus, 17α-HSD could also catalyze the transformation of DHT, 5α-dione, and 5α-pregnane-3,20-dione (DHP) into 3α-diol, ADT and 5α-pregnane-3α-ol-20-one (allopregnanolone) through its less potent 3α-HSD activity. We also have over-expressed the 17α-HSD in *Escherichia coli *and have purified it by affinity chromatography. The purified enzyme exhibits the same catalytic properties that have been observed with cultured HEK-293 stably transfected cells. Using quantitative Realtime-PCR to study tissue distribution of this enzyme in the mouse, we observed that it is expressed at very high levels in the kidney.

**Conclusion:**

The present study permits to clarify the biosynthesis pathway of epiT. It also offers the opportunity to study gene regulation and function of this enzyme. Further study in human will allow a better comprehension about the use of epiT in drug abuse testing; it will also help to clarify the importance of its accumulation in breast cyst fluid and prostate, as well as its potential role as natural antiandrogen.

## Background

Epitestosterone (17α-hydroxy-4-androstene-3-one) is an epimer of testosterone (T). Its concentration in the urine is used as reference substance in the control of T abuse [1]. EpiT was identified for the first time as an androgen metabolite produced by rabbit liver slices [2]. It has been also observed that slices of rabbit, guinea pig and dog liver, mare's ovary, ox and sheep blood as well as guinea pig kidney, ovary and testis possess the ability to produce epiT from T and 4-dione [3]. In the mouse, the kidney is a major site of epiT formation, while the production in the liver is negligible [4]. In castrated male bovine, it has been observed that blood and liver both possess a high ability to convert T into epiT [5]. No interconversion of T and epiT has been observed in testes of bulls, rabbits or rats, even though it has been found that testes is a source of endogenous epiT in these species [3].

In young boys, the concentration of epiT is higher than T, however it declines in adulthood to a epiT/T ratio of approximately 1 [6]. In hyperplasic prostate, epiT concentration is comparable to that of 4-dione, which represents about twice the amount of T and half the concentration of dihydrotestosterone (DHT) [7]. The excretion of epiT in urine is slightly lower than that of T [8-11]. Plasma concentrations of epiT decline with age and were established at approximately 2.5 nmol/l in adult men and 1.2 nmol/l in women. Since epiT does not originate from endogenous T, and because the ratio of urinary T to epiT in adults is almost constant, this ratio has been used as a basis for the detection of exogenously administered T : the median ratio in normal healthy men is about 1, while being significantly elevated in case of testosterone abuse. Urinary T/epiT ratio from 1 to 6 is considered normal by the International Olympic Committee, while one displaying a ratio greater than 6 is suspected of anabolic steroid use [12]. In order to apply this assay, it is important to assume that the biosynthesis of epitestosterone does not originate from testosterone, and that both epimers undergo similar clearance. It is also important to assume that racial or individual variations do not affect this ratio. Because of these important assumptions, parameters that may influence this ratio and possibly lead to false positive results have been intensively debated since the introduction of the T/epiT ratio in doping analysis [13]. The lack of knowledge about the gene encoding the enzyme responsible for the formation of epiT has made the identification of appropriate parameters difficult.

In humans, the interconversion of T and epiT is negligible [14]. Using labeled-T, it has been shown that epiT does not originate from T. Studying the 16-ene-synthetase reaction in human testicular homogenates, Weusten et al. [15] hypothesized that 5-androstene-3β,17α-diol (epi5-diol) and 5,16-androstadien-3β-ol are synthesized from pregnenolone in a single step through a 16-ene-synthase, and then that epi5-diol is further converted to epiT by 3β-hydroxysteroid dehydrogenase (3β-HSD). On the other hand, a recent study showed that administration of 4-dione to healthy male subjects increases the urinary excretion rates of epiT, thus suggesting that epiT could be biosynthesized from 4-dione [16]. The authors suggested that a likely candidate to convert 4-dione to epiT would be 17α-hydroxysteroid dehydrogenase (17α-HSD). This enzyme has been purified from liver and kidney tissue from rabbits and hamsters [17-19]. Accordingly, in this report, we describe the isolation and characterization of a cDNA encoding an enzyme exhibiting a strong 17α-HSD acivity, efficiently catalyzing the conversion of 4-dione into epiT.

## Results

### Characterization of mouse 17α-HSD cDNA and gene

**Figure 1:**
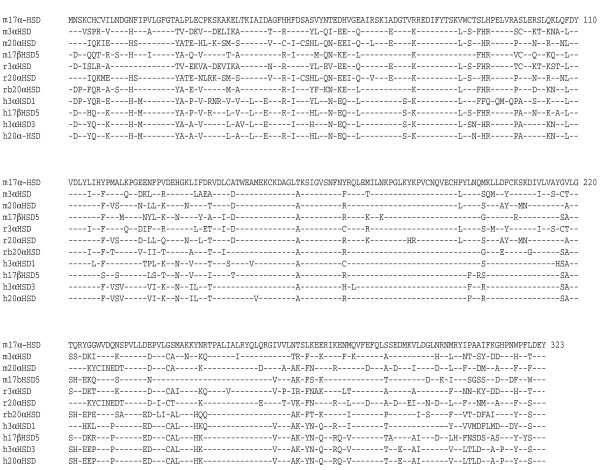
**Alignment of the amino acid sequence of mouse 17α-HSD with those of related enzymes. **Amino acid sequence of mouse 17α-HSD was aligned with sequences of mouse (m), rat (r), rabbit (rb) and human (h) enzyme members of the AKR1c subfamily. The corresponding name according to the nomenclature for aldo-keto reductase (AKR) family members are: m17α-HSD, AKR1C21; m3α-HSD, AKR1C14; m20α-HSD, AKR1C18; m17β-HSD5, AKR1C6; r3α-HSD, AKR1C9; r20α-HSD, AKR1C8; h3α-HSD1, AKR1C4; h3α-HSD3, AKR1C2; h17β-HSD5 or 3α-HSD2, AKR1C3; and h20α-HSD, AKR1C1. Amino acids are given in conventional single letter code and numbered on the right. Dashes and dots, respectively, represent identical and missing amino acids.

The mouse genome project has made available the sequence of a cluster containing eight members of the aldo-keto reductase family located in the chromosome 13 [20]. Using specific primers, we have cloned a cDNA fragment containing the entire coding region of the gene identified as AKR1C21 without notification of any activity. Sequence analysis of the cDNA fragment shows that it encodes a putative protein of 323 amino acids having a calculated MW of 36745 Daltons. We have deposited the sequence in GenBank under the accession number AY742217. Comparison of the deduced amino acid sequence with that of other aldo-keto reductase members (Figure 1) shows that mouse 17α-HSD shares 77, 70 and 72 % amino acid sequence identity with mouse type 5 17β-HSD, 3α-HSD and 20α-HSD respectively. It also shares 70, 69, 71, 72, 72, 73, 74 % with rat 3α-HSD and 20α-HSD, rabbit 20α-HSD as well as human 3α-HSD1, 17β-HSD5, 3α-HSD3 and 20α-HSD. The genomic structure as derived from public data bank indicates that mouse 17α-HSD gene is 12.5 kb long covering 9 exons and is transcribed into a 1.2 kb mRNA.

### Identification of epiT by HPLC

**Figure 2:**
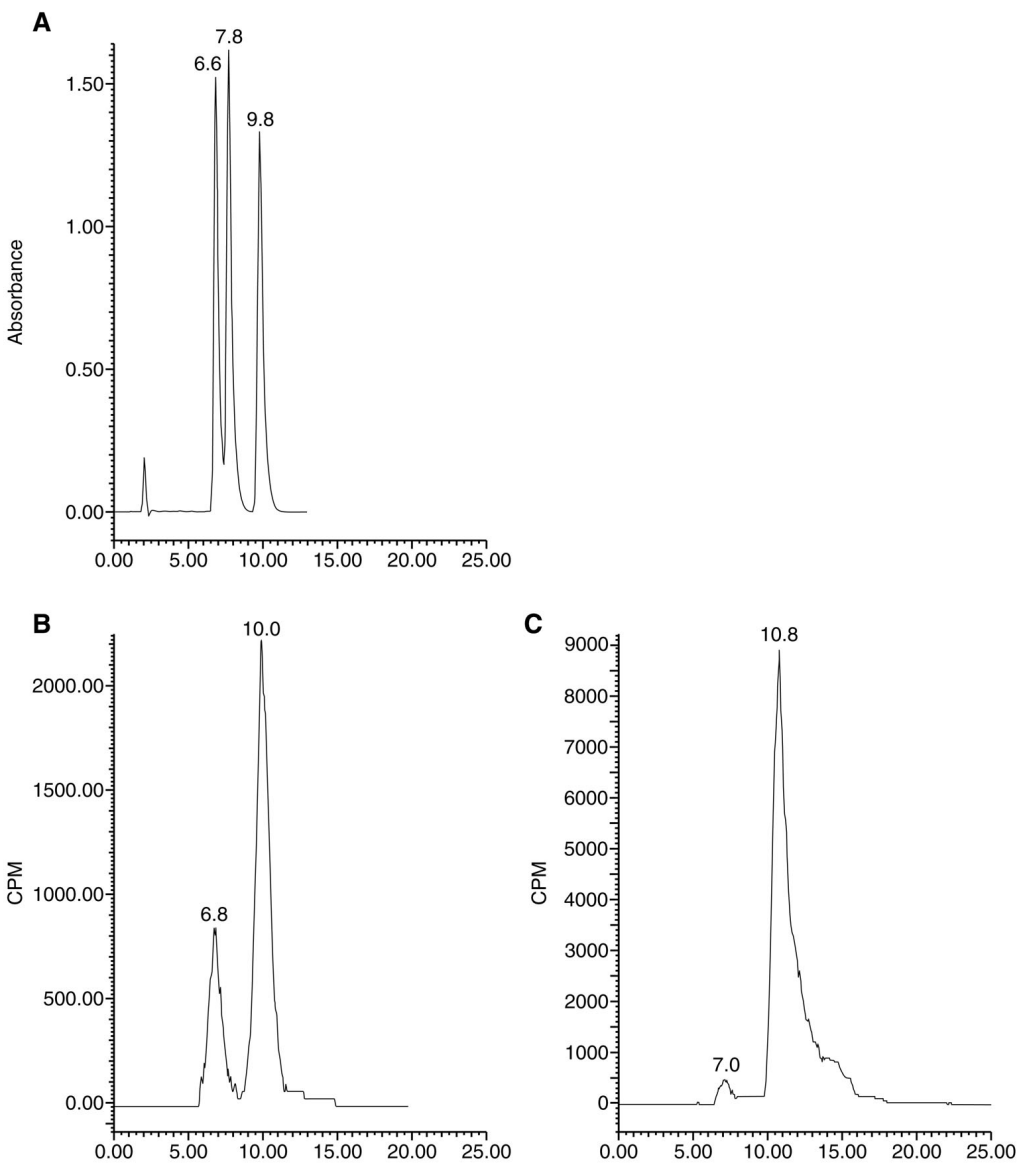
**Identification by HPLC of epiT produced from HEK-293 cells stably expressing 17α-HSD. **(A) Elution profile of non labeled steroids; 4-dione (1^st ^peak), testosterone (2^nd ^peak) and epitestosterone (3^rd ^peak). (B) Products extracted from enzymatic assay done with cells stably expressing 17α-HSD, substrate is 4-dione. (C) Products extracted from enzymatic assay done with purified enzyme; substrate is 4-dione. Separation and identification of metabolites were performed as described in *Materials and Methods*.

**Figure 3:**
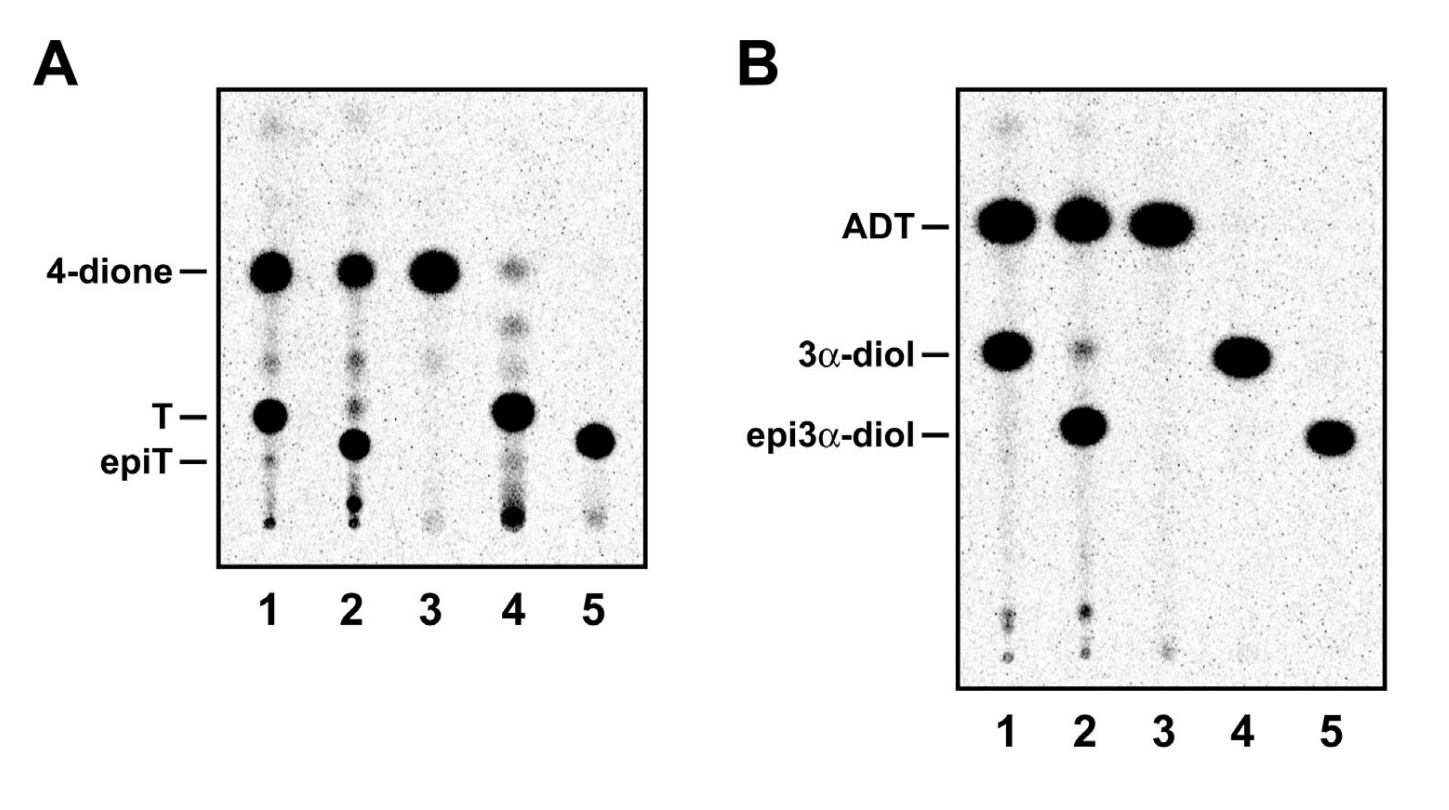
**Identification of the 17α-HSD activity by TLC. **A- Incubation of [^14^C]-4-dione with cells stably expressing mouse 17β-HSD type 5 (1) and 17α-HSD (2) activity. Standards of 4-dione (3), T (4) and epiT (5) have been deposited and co-migrated. B- Incubation of [^14^C]-ADT with cells cells stably expressing mouse 17β-HSD type 5 (1) and 17α-HSD (2) activity. Standards of ADT (3), 3α-diol (4) and epi3α-diol (5) have been deposited and co-migrated.

Preliminary data using [^14^C]4-dione as substrate showed that the product resulting from the transformation of 4-dione by the enzyme overexpressed in HEK-293 cells is different from T, the metabolite produced by a 17β-HSD activity. To verify the identity of this metabolite, we used HPLC to analyze extracts of culture medium of HEK-293 cells over expressing 17α-HSD in presence of [^14^C]4-dione (Figure 2B). We also analyzed metabolites obtained after [^14^C]4-dione incubation with the pure enzyme (Figure 2C). Comparison of the HPLC elution and TLC migration profile of the product with a commercial epiT standard clearly indicates that the product is epitestosterone, the 17α-epimer of testosterone. Using TLC separation, we were also able to verify the identity of epi3α-diol, which is produced from ADT, in addition of epitestosterone In order to confirm the 17α-HSD nature of the activity, comparison of the products obtained from incubation of 4-dione (Figure 3A) and ADT (Figure 3B) with 17β-HSD type 5 and 17α-HSD was accomplished.

### Substrate specificity of the mouse 17α-HSD activity

**Figure 4:**
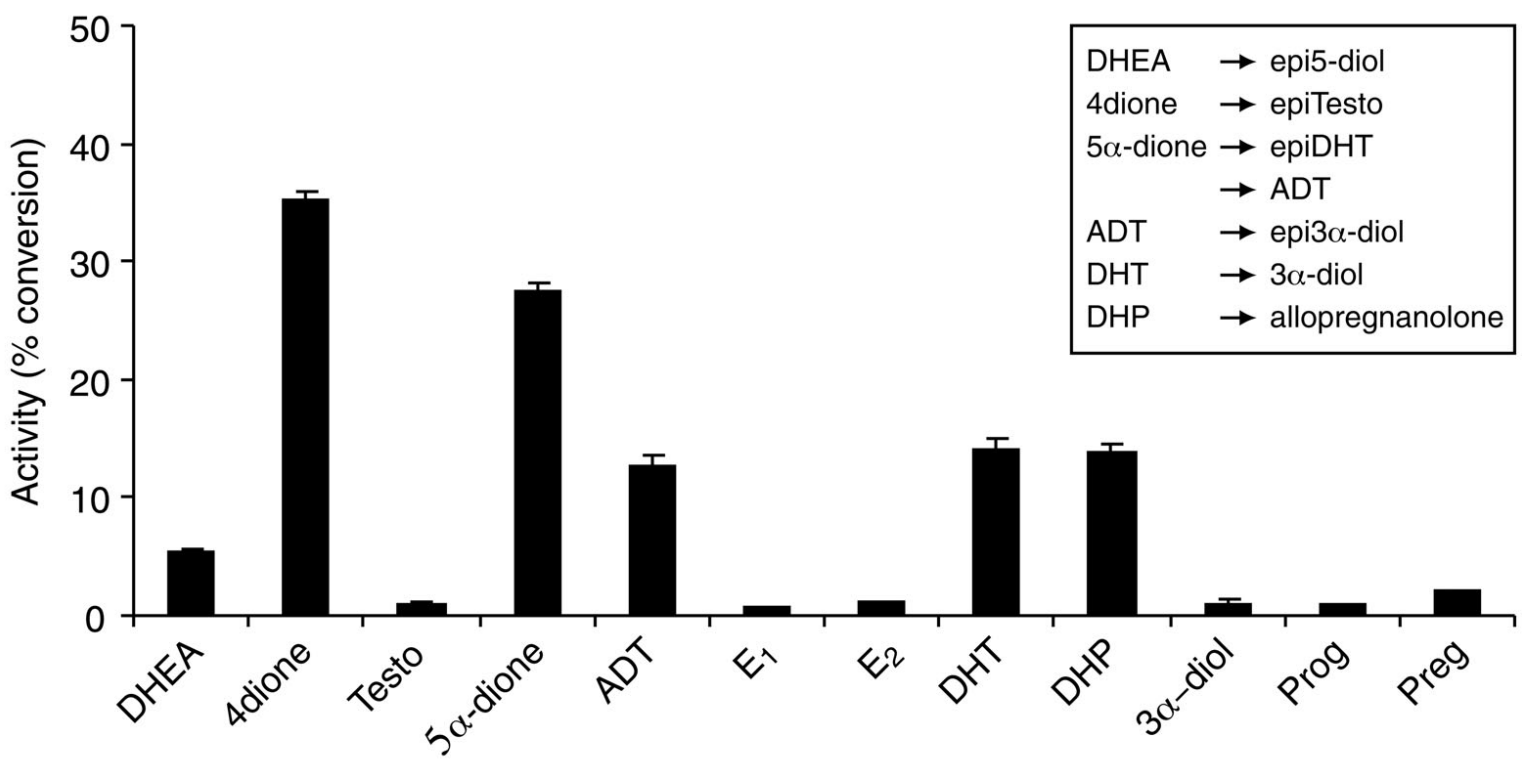
**Substrate specificity of mouse 17α-HSD activity of HEK-293 cells stably transfected with pCMVneo-m17α-HSD. **The experiments were performed using HEK-293 cells stably expressing 17α-HSD in culture. 0,1 μM of the indicated [^14^C]-and [^3^H]-labeled steroid was added to culture medium for one hour. Testo, conversion of testosterone to 4-dione and vice-versa; E1, conversion of estrone to estradiol and vice-versa; DHEA, conversion of dehydroepiandrosterone to 5-diol; 5α-dione, conversion of androstanedione to androsterone (ADT) and vice-versa; DHT, conversion of dihydrotestosterone to 3α-diol and vice-versa; DHP, conversion of dihydroprogesterone to allopregnanolone and vice-versa; Preg and Prog, conversion of pregnenolone and progesterone to 20α-OHPreg and 20α-OHProg. The error bar indicates mean ± SEM of triplicate assays. Incubation, extraction, separation and quantification were performed as described in *Materials and Methods*.

Using 17α-HSD stably expressed in HEK-293 cells, we have characterized the substrate specificity of this enzyme in cultured cells by comparison with non transfected cells. As illustrated in Figure 4 and Table 1, mouse 17α-HSD possesses two activities. The strongest activity is 17α-reductase activity responsible for the transformation of 4-dione into epiT as well as the transformation of ADT, 5α-dione and DHEA into epi3α-diol, epiDHT and epi5-diol, respectively. The second activity is 3α-reductase, responsible for the transformation of DHT to 3α-diol, 5α-dione to ADT and DHP to allopregnanolone. Moreover, we found that purified enzyme displays the same catalytic activity as the overexpressed enzyme in intact cells.

**Table 1:** Kinetic constants of 17α-HSD for various substrates

	**K_m _**(μM)	**V_max _**(μmol/min)	**V_max _/ K_m_**
**4-dione**	0,4	0,3	0,7
**ADT**	0,9	0,3	0,3
**DHEA**	3,5	0,4	0,1
**DHT**	2,0	0,7	0,3

### Tissue distribution of mouse 17α-HSD

Using Q_RT-PCR to quantify mRNA expression levels of 17α-HSD in male and female (Table 2) mouse tissues, we found that the expression of this enzyme is highly specific for the mouse kidney, while type 5 17β-HSD, the enzyme catalyzing the formation of T, is markedly expressed in the liver. Both enzymes are expressed at very high levels, more than 15 millions copies/μg total RNA. Since the estimated amount of total RNA in a single liver cell is about 50 pg [21], we consider that an expression level of 20,000 copies/μg total RNA corresponds to approximately 1 copy/cell in an homogenous cell population. Therefore, 15 millions copies/μg total RNA should correspond to 750 copies/cell.

**Table 2:** Quantification of mRNA expression levels of 17α-HSD and 17β-HSD5 in various mouse tissues using Realtime PCR

	**17α-HSD **(number of copies/μg RNA ± SEM)		**17β-HSD5 **(number of copies/μg RNA ± SEM)	
	
	**male**	**female**	**male**	**female**
**Pituitary gland**	130 ± 92	692 ± 251	572 ± 992	2986 ± 2591
**Adrenal**	0 ± 0	12036 ± 356	53445 ± 11410	3714 ± 1960
**Liver**	2344 ± 6	332 ± 73	15369159 ± 2701597	14995082 ± 987906
**Kidney**	22226386 ± 1068282	17083578 ± 928740	45536 ± 9194	102586 ± 35774
**Spleen**	21610 ± 1369	7466 ± 438	22791 ± 4358	8777 ± 5673
**Lung**	0 ± 0	0 ± 0	0 ± 0	6097 ± 2642
**Thymus**	2029 ± 40	3790 ± 134	407 ± 705	10943 ± 7200
**Stomach**	0 ± 0	0 ± 0	11858 ± 4062	8539 ± 4606
**Colon**	13860 ± 2184	6299 ± 1049	256686 ± 37628	17438 ± 21120
**Heart**	0 ± 0	0 ± 0	0 ± 0	6821 ± 4402
**Testis**	6333 ± 490	-	0 ± 0	-
**Prostate**	0 ± 0	-	1095 ± 1896	-
**Preputial gland**	2681 ± 261	-	14551 ± 12605	-
**Ovary**	-	1980 ± 120	-	7208 ± 6397
**Uterus**	-	0 ± 0	-	8328 ± 3819
**Clitoral gland**	-	6516 ± 337	-	14419 ± 12591
**Mammary gland**	-	0 ± 0	-	5666 ± 3188

## Discussion

The present report describes the isolation and characterization of a cDNA sequence encoding the enzyme 17α-HSD. We have shown that this enzyme converts mainly 17-ketosteroids into 17α-hydroxysteroids : 4-dione to epiT, ADT to epi3α-diol, 5α-dione to epiDHT and DHEA to epi5-diol. Since the enzyme is able to catalyze 4 distincts 17-ketosteroids into its 17α-hydroxy-compounds, and because all the four products have shown identitical profile on HPLC and TLC with commercial products, the results, thus, represent a strong evidence that the activity catalyzed by this specific enzyme is a 17-ketoreductase activity producing 17α-hydroxysteroids.

17α-HSD belongs to the aldo-keto reductase family and shares 77 % amino acid with the mouse type 5 17β-HSD, an enzyme catalyzing the transformation of 4-dione into T [22]. To our knowledge, this is the first example of two highly homologous enzymes belonging to the same gene family, and catalyzing the formation of two distinct epimers from a same substrate. This will represent an interesting model to study the mechanism of the 17α- and 17β- stereospecificity. In addition to the difference in substrate specificity, mouse 17α-HSD and 17β-HSD5 show distinct and specific mRNA tissue distribution : 17α-HSD is highly expressed in kidney while 17β-HSD5 is abundant in the liver. This is in agreement with previous reports showing that mouse liver does not produce epiT [3].

We have previously shown that mouse type 5 17β-HSD is specifically expressed in the liver [22], while human type 5 17β-HSD is more widely expressed [23]. It is noteworthy that 17α-HSD and type 5 17β-HSD belongs to aldo-keto reductase family. Members of this family share very high homology although they catalyze different activities and are expressed in different tissues; for example, mouse type 5 17β-HSD shares 77, 88 and 86 % identity with mouse 17α-HSD, 3α-HSD and 20α-HSD respectively. Therefore, previous studies based on hybridation experiments such as northern blot analysis could not distinguish different members of the aldo-keto-reductase family. Using Realtime-PCR with specific primers, as described in the present manuscript, mouse type 5 17β-HSD could be specifically identified, distinctly from 17α-HSD.

**Figure 5:**
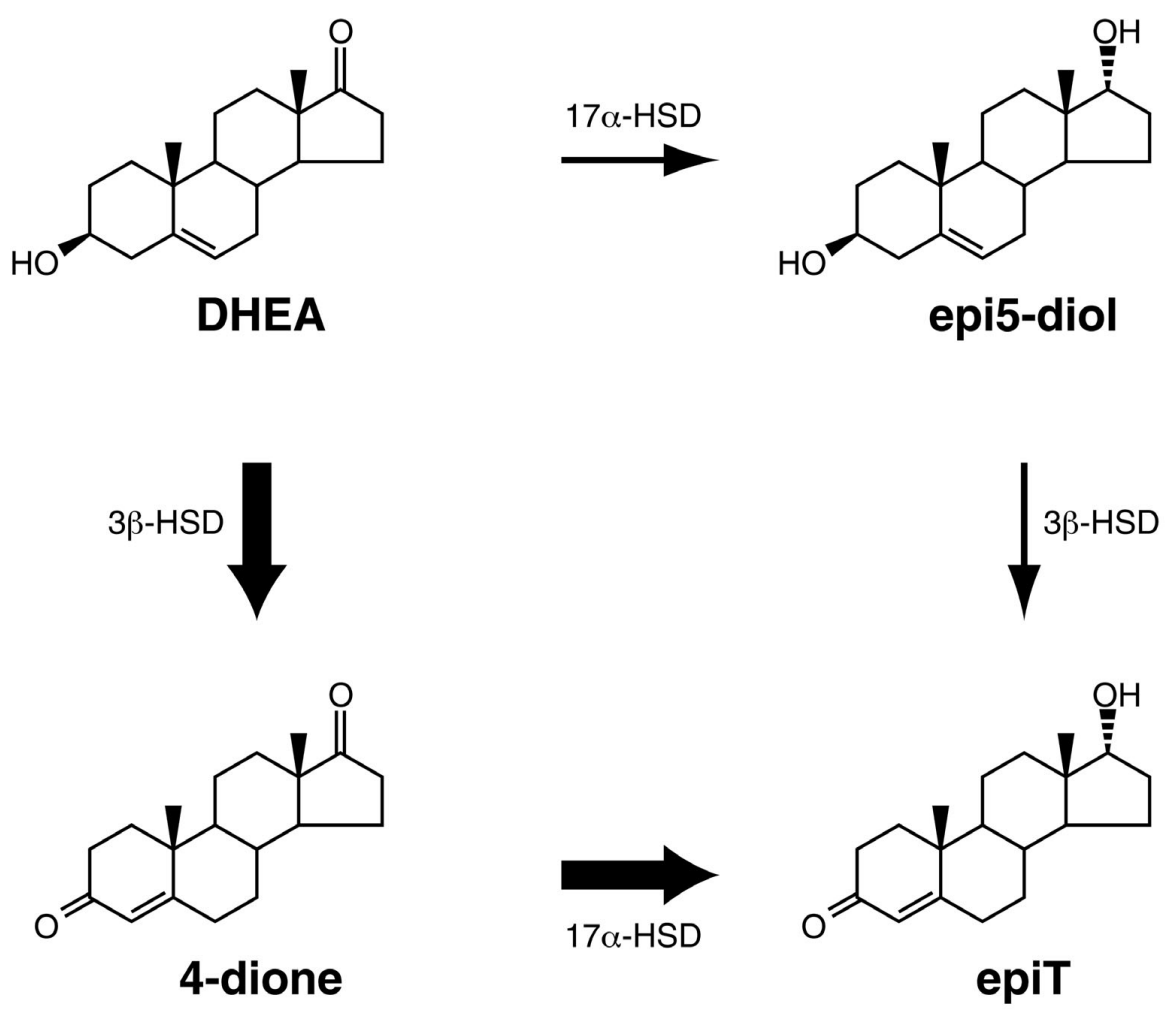
**Diagram illustrating the 2 putative pathways for the conversion of DHEA to epiT via 17α-HSD and 3β-HSD. **The thickness of the arrows indicates the relative importance of each pathway

Our results show that 17α-HSD is able to convert 4-dione to epiT as well as DHEA to epi5α-diol, and thus suggest that epiT could be produced through two different pathways involving 17α-HSD (Figure 5). However, the catalytic efficiency of 3β-HSD and 17α-HSD for DHEA and 4-dione respectively, strongly suggest that the main pathway leading to the formation of epiT is the conversion of DHEA by 3β-HSD and the 4-dione product being further converted into epiT. In a previous study described by Weusten et al. [15], it was shown that a non-negligible quantity of epi5-diol is produced by 16-ene-synthase activity, the reaction catalyzing the transformation of pregnenolone into 5,16-androstadien-3β-ol. The authors thus suggested that epi5-diol could be produced directly from pregnenolone, and epiT being produced by the subsequent conversion of epi5-diol by 3β-HSD. In contrast, our data strongly suggests that the epi5-diol originates from the transformation of pregnenolone to DHEA by P450c17, followed by the conversion of DHEA into epi5-diol by the enzyme 17α-HSD. Previously, we have shown that P450c17 possesses two activities, 17α-hydroxylase/17-20 lyase and 16-ene synthase, that are able to convert pregnenolone into DHEA and 5,16-androstadien-3β-ol, respectively [24]; however, no epi5-diol has been detected.

The present first report of 17α-HSD will help to better understand the physiological role of epiT. It offers a tool to further study at molecular level the role of 17α-HSD in human, espectially for T abuse testing in sports using epiT as a control. It also permits to investigate the importance of epitestosterone in some previously reported interesting phenomenons such as its accumulation in mammary cyst fluid [25] and in human prostate [7,26], as well as its potential neuroprotective effects [27], and natural anti-androgenic effects [28].

## Methods

### Isolation of mouse 17α-HSD

A cDNA fragment of a coding region of mouse 17α-HSD (AKR1C21) was amplified by polymerase chain reaction (PCR) from a mouse spleen cDNA and the oligoprimer pair (5'-ggg-gtc-gac-ttt-gaa-gag-gga-cac-ata-atg-a-3' and 5'-ggg-ggt-acc-acc-cat-agg-ctt-ttc-agg-aga-3') derived from the DNA sequence NM_029901 from GenBank database. Mouse spleen cDNA was obtained by reverse transcription of 20 μg of mouse spleen total RNA using 400 U SuperScript II reverse transcriptase (Invitrogen, Burlington, Ontario, Canada) and oligo-d(T)24 as primer in a reaction buffer containing 50 mM Tris-HCl pH 8.3, 75 mM KCl, 3 mM MgCl_2_, 10 mM DTT and 0.5 mM dNTPs. The resulting cDNA fragments were subcloned into a pCMVneo expression vector (pCMVneo-m17α-HSD) which was subsequently used to produce a stably transfected HEK-293 cell line. Plasmid DNA was prepared using the Qiagen Mega Kit (Qiagen, Chatsworth, CA, USA). Sequence of the pCMVneo-m17α-HSD was determined using an ABI 3730/XL automatic sequencer, to verify the identity of the amplified sequence.

### Stable expression in HEK-293 cells

Stable transfection of pCMVneo-m17α-HSD into HEK-293 cells was performed as described previously [29]. Briefly, HEK-293 cells were cultured in 6-well falcon flasks to approximately 3 × 10^5 ^cells/well in Minimum Essential Medium (MEM) (Invitrogen) supplemented with 10% (vol/vol) FCS (Wisent, Saint-Bruno, Québec, Canada) at 37°C under a 95% air- 5% CO_2 _humidified atmosphere. Five μg of pCMVneo-m17α-HSD was transfected using Exgen 500 reagent (Fermentas, Burlington, Ontario, Canada). After 6 h incubation at 37°C, the transfection medium was removed and 2 ml of MEM were added. Cells were further cultured for 48 h, then transferred into 10 cm Petri dishes and cultured in MEM containing 700 μg/ml of G-418 (Invitrogen) in order to inhibit the growth of non-transfected cells. The medium containing G-418 was changed every two days until resistant colonies were observed.

### Overexpression and purification of mouse 17α-HSD

The cDNA encoding mouse 17α-HSD was subcloned into a pGEX vector (Amhersham Biosciences, Baie d'Urfé, Québec, Canada) and expressed in *Escherichia coli *BL21(DE3) pLysS as a fusion protein with glutathione-S-transferase (GST). The fusion protein was isolated using a Glutathione-Sepharose 4B column, as described by the manufacturer. The purified 17α-HSD enzyme was separated from GST by digestion with thrombin. 17α-HSD is not adsorbed on the DEAE column and is recuperated in the flow-through fraction, while GST and fusion protein remain on the column. With this method we obtain about 10 mg of a purified enzyme preparation per 100 ml of cell culture. Analysis of samples obtained during the purification process was performed using sodium dodecyl sulfate polyacrylamide gel electrophoresis (SDS-PAGE), as described before [30]. The broad range molecular weight standards was purchased from Bio-Rad (Missisauga, Ontario, Canada).

### Assay of enzymatic activity

Enzymatic activities of 17α-HSD were determined using both purified enzyme and the cultured HEK-293 cells stably transfected with pCMVneo-m17α-HSD, as previously described [29]. Briefly, 3 μg of purified 17α-HSD were incubated with 10 mM NADPH, 0.1 μM of 4-dione in phosphate saline buffer, 50 mM, pH 7.3, for 20 minutes. For the intact cells, 0.1 μM of the [^14^C]-labeled steroid (PerkinElmer, Boston, Massachussetts, USA) was added to freshly changed culture medium in a 6-well culture plate. Non-transfected HEK-293 cells were used as control of the background. After incubation, the steroids were extracted with 2 ml of ether. The organic phases were pooled and evaporated to dryness. The steroids were then solubilized in 50 μl of dichloromethane, applied to Silica gel 60 thin layer chromatography (TLC) plates (Merck, Darmstad, Germany). To obtain a better separation and identification of metabolites, different solvent systems were used. Metabolites of substrates 4-dione, DHEA and 5α-dione were separated in chloroform : ether (9:1), while DHP, DHT and ADT products were separated in the toluene : acetone (4:1) solvent system. Substrates and metabolites were identified by comparison with reference steroids, revealed by autoradiography and quantified using the PhosphorImager System (Molecular Dynamics, Sunnyvale, California, USA). The enzymatic reaction was carried out using the condition in which the activity varies linearly with the enzyme concentration and incubation time, indicating that the cofactor concentration produced by the cells is in excess; the reverse reaction was consequently prevented. In our conditions, this linearity was observed at even more than 60% transformation. Determination of the kinetic parameters was done by Lineweaver-Burk graph analysis using Enzfitter software.

### Identification of epitestosterone by High Performance Liquid Chromatography (HPLC)

^14^C-Labeled steroids were analyzed using Waters NovaPak reverse-phase C18 HPLC column (3.9 × 150 mm, 4 μm). The mobile phase was MeOH/H_2_O/THF (26 : 56 : 18 v/v), with a flow rate of 0.7 ml.min^-1^. Radioactivity was monitored in the eluent using Beckman 171 HPLC Radioactivity Monitoring System. Unlabelled steroids (4-dione, DHEA, 5α-dione, ADT, T, 5-diol, DHT, 3α-diol, epiT, epi5-diol, epiDHT and epi3α-diol) were obtained from Steraloids (Newport, Rhode Island, USA) and used as standards.

### Tissue collection and RNA preparation

Total RNA of indicated tissues was isolated using Trizol Reagent (Invitrogen, Burlington, Ontario) as described by the manufacturer. Twenty μg of total RNA was converted to cDNA by incubation at 42°C for 1 h with 400 U SuperScript II reverse transcriptase (Invitrogen), using oligo-d(T)24 as primer in a reaction buffer containing 50 mM Tris-HCl pH 8.3, 75 mM KCl, 3 mM MgCl_2_, 10 mM DTT and 0.5 mM dNTPs. The tissues were collected in C57BL6 mice at 12-15 weeks of age obtained from Charles River, Inc. (Saint-Constant, Québec, Canada). The mice were housed individually in vinyl cages. The photoperiod was 12 h of light and 12 h of darkness (lights on at 07:15 h). Certified rodent food (Lab Rodent Diet) and tap water were provided *ad libitum*. The experiment was conducted in an animal facility approved by the Canadian Council on Animal Care (CCAC) and the Association for Assessment and Accreditation of Laboratory Animal Care (AAALAC). The study was performed in accordance with the CCAC Guide for Care and Use of Experimental Animals. The collected organs were rapidly trimmed, snap-frozen in liquid nitrogen and stored at -80°C until RNA extraction.

### Tissue distribution of 17α-HSD mRNA using RealTime PCR

Total RNA from pituitary gland, adrenal, liver, kidney, spleen, thymus, stomach, heart, lung, ovary, uterus, clitoral gland, mammary gland, testis, prostate and preputial gland, prepared as described above, were analyzed for the expression of 17α-HSD mRNA using quantitative RealTime RT-PCR (Q_RTPCR). cDNA corresponding to 20 pg of the initial total RNA was used to perform fluorescent-based Realtime PCR quantification using the LightCycler Realtime PCR apparatus (Roche Inc. Nutley, NJ). Reagents were obtained from the same company and were used as described by the manufacturer. The conditions for the PCR reactions were: denaturation at 95°C for 10 sec, annealing at 62°C for 5 sec and elongation at 72°C for 8 sec. Oligoprimer pairs (5'-ttg-att-gcc-ctt-cgc-tac-cag-3', 5'-aaa-tgg-cag-cag-gta-tgt-atc-gc-3') allowed the amplification of approximately 170 bp of the mouse 17α-HSD sequence. Data calculation and normalization was performed using second derivative and double correction method as previously described [31]. 17α-HSD mRNA expression levels are expressed as number of copies/μg total RNA using a standard curve of Cp versus logarithm of the quantity. The standard curve was established using known cDNA amounts of 0, 10^2^, 10^3^, 10^4^, 10^5 ^and 10^6 ^copies of cDNA and a LightCycler 3.5 program provided by the manufacturer (Roche Inc).

## Abbreviations

T testosterone

epiT epitestosterone

4-dione androstenedione

epi5-diol 5-androstene-3α,17α-diol

ADT androsterone

DHEA dehydroepiandrosterone

5α-dione androstanedione

DHP 5α-pregnane-3,20-dione

DHT 5α-dihydrotestosterone

epi3α-diol 5α-androstane-3α,17α-diol

5α-pregnane-3α-ol-20-one allopregnanolone

3α-diol 5α-androstane-3α,17α-diol

5-diol 5-androstene-3β,17α-diol

epiDHT 5α-androstane-3-one-17α-ol

HSD hydroxysteroid dehydrogenase

PCR polymerase chain reaction

## Authors' contributions

VB has participated in the design of the study and in redaction of the manuscript; she has carried out the molecular biology manipulations, all enzymatic assays, and everything surrounding the culture of the cells. FF has taken care of the entire process of enzyme's purification. RB has participated in the conception of the study, especially the purified enzyme part. VLT conceived the study and was implicated in the redaction of the article. All authors read and approved final manuscript.

**Figure 6:**
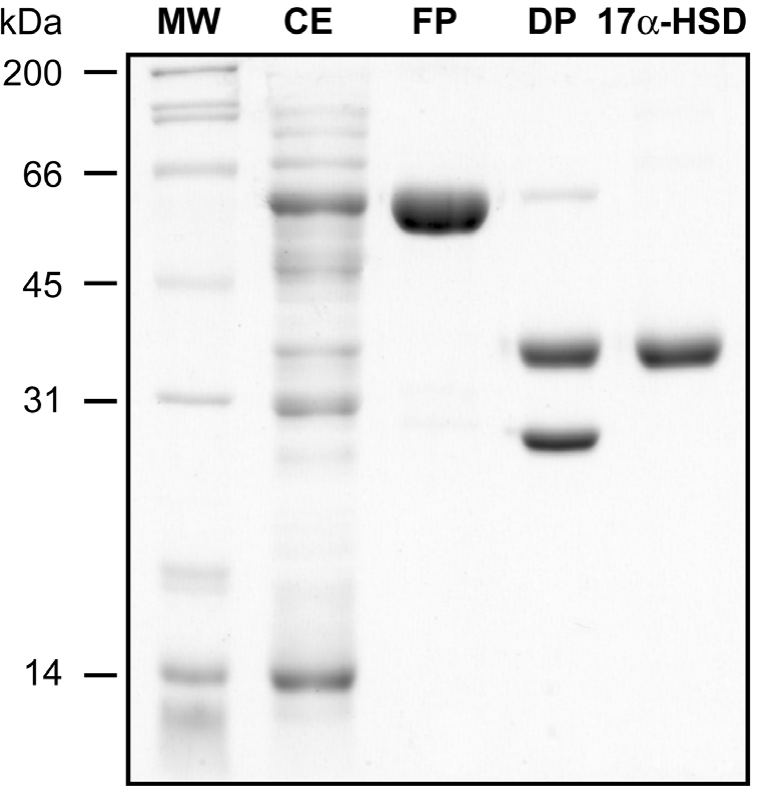
**SDS-PAGE of fractions obtained during the purification process of 17α-HSD. **MW, molecular weight standards; CE, cell extract of 100000 g; FP, fusion protein; DP, protein obtained after digestion with thrombin; 17α-HSD, purified.

## References

[B1] Kuoppasalmi K, Karjalainen U, Tehunen R (1984). Doping analysis in Helsinki 1983. Clinical Chemistry Research Foundation Publications.

[B2] Clark LC, Kochakian CD (1947). The in vitro metabolism of testosterone to 4-androstenedione-3,17 cis-testosterone and other steroids by rabbit liver slices. Journal of biological chemistry.

[B3] Starka L (2003). Epitestosterone. J Steroid Biochem Mol Biol.

[B4] Arimasa N, Kochakian CD (1973). Epitestosterone and 5alpha-androstane-3alpha,17beta-diol: the characteristic metabolites of androst-4-ene-3,17-dione produced by mouse kidney in vitro. Endocrinology.

[B5] Martin RP (1966). Fecal metabolites of testosterone-4-14C in the bovine male castrate. Endocrinology.

[B6] Lapcik O, Hampl R, Hill M, Starka L (1995). Plasma levels of epitestosterone from prepuberty to adult life. J Steroid Biochem Mol Biol.

[B7] Starka L, Hampl R, Hill M, Lapcik O, Bilek R, Petrik R (1997). Epitestosterone in human blood and prostatic tissue. Eur J Clin Chem Clin Biochem.

[B8] De Nicola AF, Dorfman RI, Forchielli E (1966). Urinary excretion of epitestosterone and testosterone in normal individuals and hirsute and virilized females. Steroids.

[B9] Bilek R, Hampl R, Putz Z, Starka L (1987). Radioimmunoassay of epitestosterone: methodology, thermodynamic aspects and applications. J Steroid Biochem.

[B10] France JT, Knox BS (1967). Urinary excretion of testosterone and epitestosterone in hirsutism. Acta Endocrinol (Copenh).

[B11] Longhino N, Tajic M, Vedris M, Jankovic D, Drobnjak P (1968). Urinary excretion of androstenedione, testosterone, epitestosterone and dehydroepiandrosterone during the normal menstrual cycle. Acta Endocrinol (Copenh).

[B12] Donike M, Barwald KR, Klostermann K, Schanzer W, Zimmermann J, Heck H, Hollmann W and Liesen H (1983). Nachweis von exogenem testosteron in sport. Leistung und Gesendheit.

[B13] Dehennin L, Peres G (1996). Plasma and urinary markers of oral testosterone misuse by healthy men in presence of masking epitestosterone administration. Int J Sports Med.

[B14] Dray F, Ledru MJ (1966). [Metabolism of epitestosterone. Absence of peripheral interconversion of epitestosterone and testosterone and existence of a production of epitestosterone sulfate in normal adult men]. C R Acad Sci Hebd Seances Acad Sci D.

[B15] Weusten JJ, Legemaat G, van der Wouw MP, Smals AG, Kloppenborg PW, Benraad T (1989). The mechanism of the synthesis of 16-androstenes in human testicular homogenates. J Steroid Biochem.

[B16] Catlin DH, Leder BZ, Ahrens BD, Hatton CK, Finkelstein JS (2002). Effects of androstenedione administration on epitestosterone metabolism in men. Steroids.

[B17] Kochakian CD (1982). 17 alpha and 17 beta-oxidoreductases of adult female hamster liver and kidney. J Steroid Biochem.

[B18] Lau PC, Layne DS, Williamson DG (1982). Comparison of the multiple forms of the soluble 3(17) alpha-hydroxysteroid dehydrogenases of female rabbit kidney and liver. J Biol Chem.

[B19] Hasnain S, Williamson DG (1977). Properties of the multiple forms of the soluble 17alpha-hydroxy steroid dehydrogenase of rabbit liver. Biochem J.

[B20] Vergnes L, Phan J, Stolz A, Reue K (2003). A cluster of eight hydroxysteroid dehydrogenase genes belonging to the aldo-keto reductase supergene family on mouse chromosome 13. J Lipid Res.

[B21] Manual LC (2002). LightCycler presentation.

[B22] Rheault P, Charbonneau A, Luu-The V (1999). Structure and activity of the murine type 5 17beta-hydroxysteroid dehydrogenase gene(1). Biochim Biophys Acta.

[B23] Dufort I, Rheault P, Huang XF, Soucy P, Luu-The V (1999). Characteristics of a highly labile human type 5 17beta-hydroxysteroid dehydrogenase. Endocrinology.

[B24] Soucy P, Lacoste L, Luu-The V (2003). Assessment of porcine and human 16-ene-synthase, a third activity of P450c17. Eur J Biochem.

[B25] Bicikova M, Szamel I, Hill M, Tallova J, Starka L (2001). Allopregnanolone, pregnenolone sulfate, and epitestosterone in breast cyst fluid. Steroids.

[B26] Hill M, Bilek R, Safarik L, Starka L (2000). Analysis of relations between serum levels of epitestosterone, estradiol, testosterone, IGF-1 and prostatic specific antigen in men with benign prostatic hyperplasia and carcinoma of the prostate. Physiol Res.

[B27] Hammond J, Le Q, Goodyer C, Gelfand M, Trifiro M, LeBlanc A (2001). Testosterone-mediated neuroprotection through the androgen receptor in human primary neurons. J Neurochem.

[B28] Nuck BA, Lucky AW (1987). Epitestosterone: a potential new antiandrogen. J Invest Dermatol.

[B29] Huang XF, Luu-The V (2000). Molecular characterization of a first human 3(alpha-->beta)-hydroxysteroid epimerase. J Biol Chem.

[B30] Laemmli UK (1970). Cleavage of structural proteins during the assembly of the head of bacteriophage T4. Nature.

[B31] Van LT, Paquet N, Calvo E, Cumps J (2005). Improved real-time RT-PCR method for high-throughput measurements using second derivative calculation and double correction. Biotechniques.

